# Supplementary material for: The prevalence of ulnar neuropathy at the elbow and ulnar nerve dislocation in recreational wheelchair marathon athletes
Source: PLoS One. 2020 Dec 14;15(12):e0243324. doi: 10.1371/journal.pone.0243324 (PMC7735619; doi:10.1371/journal.pone.0243324)
Supplement: S1 Fig — (PDF) [file pone.0243324.s001.pdf]

Questionnaires

Name：____________________

Birthday(age)：______________ （____yrs）

Gender：__________

Body Hight：________cm Body Weight：______kg

Body Mass Index：______kg/cm^2

Primary disease（__________________________________）

Medical history（____）

History of primary disease(yrs)（______________________________________）

Q、Which is your dominant hand?

□ Right
□ Left
□ Both

Q、How long have you been in a wheelchair marathon?

( )

Q、Do you play other sports?

□ Yes
□ No
→ If yes, How long do you play?

( )

Q、 Do you have a history of wheelchair sports injuries?

□ Yes
□ No
→ If yes, what is it?

( )

Q、 How often do you practice in a week?

(times)

Q、 How long do you practice at one session?

(minuites)

Q、 How many times do you do resistance training in a week?

□ zero
□ once
□ twice
□ more than three

Q、 Do you have numbness at the elbow?

□ Yes: at only right side □ Yes: at only left side □ Yes: at both sides
□ No

Q、 How long have you had numbness at right elbow?

( )

Q、 Which part of right elbow is numb?

( )

Q、 Do you treat numbness at right elbow?

□ Yes
□ No

Q、 How long have you had numbness at left elbow?

( )

Q、 Which part of left elbow is numb?

(   )

Q、 Do you treat numbness at left elbow?

- □ Yes
- □ No
