# Supplementary material for: The prevalence of ulnar neuropathy at the elbow and ulnar nerve dislocation in recreational wheelchair marathon athletes
Source: PLoS One. 2020 Dec 14;15(12):e0243324. doi: 10.1371/journal.pone.0243324 (PMC7735619; doi:10.1371/journal.pone.0243324)
Supplement: S2 Fig — (PDF) [file pone.0243324.s002.pdf]

問診票 (Questionnaires)

名前：____________________

生年月日(年齢)：______________ (____歳)

性別：__________

身長：________cm　体重：______kg　BMI：______kg/cm^2

診断名　(__________________________________)

既往歴　(____)

罹患期間 (______________________________________)

Q、利き手はどちらですか？
□　　右
□　　左
□　　どちらでもない

Q、車いすマラソン歴はどれくらいですか？
（　　　　　　　　　　　　　　　　　　　　　）

Q、他のスポーツはしていますか？
□　　はい
□　　いいえ
→はいの場合それは何ですか？
（　　　　　　　　　　　　　　　　　　　　　）
→はいの場合いつからしています？
（　　　　　　　　　　　　　　　　　　　　　）

Q、車いすスポーツ外傷歴はありますか？
□　　はい
□　　いいえ
→はいの場合それは何ですか？
（　　　　　　　　　　　　　　　　　　　　　）

Q、練習頻度はどれくらいですか？
（週に　　　　　　　　　　　　　　回　）

Q、一回の練習時間はどれくらいですか？
（　　　　　　　　　　　　　　　　　分　）

Q、 筋力トレーニングはどの程度実施していますか？
□　　全くしない
□　　週一回
□　　週二回
□　　週三回以上

Q、肘のしびれはありますか？
□　　右肘のみ　□　　左肘のみ　□　　両肘
□　　両肘ない

Q、右肘はいつからしびれていますか？
（　　　　　　　　　　　　　　　　　　　　　）

Q、右肘のどこがしびれますか？
（　　　　　　　　　　　　　　　　　　　　　）

Q、右肘しびれの治療はしていますか？
□　　はい
□　　いいえ

Q、左肘はいつからしびれていますか？
（　　　　　　　　　　　　　　　　　　　　　　　　　　）

Q、左肘のどこが痛みますか？
（　　　　　　　　　　　　　　　　　　　　　　　　　　）

Q、左肘しびれの治療はしていますか？
□　　はい
□　　いいえ
