# Supplementary material for: The prevalence of ulnar neuropathy at the elbow and ulnar nerve dislocation in recreational wheelchair marathon athletes
Source: PLoS One. 2020 Dec 14;15(12):e0243324. doi: 10.1371/journal.pone.0243324 (PMC7735619; doi:10.1371/journal.pone.0243324)
Supplement: S1 Table — (PDF) [file pone.0243324.s003.pdf]

age(yrs)	gender	body hight(cm)	body weight(Kg	BMI	primary disea	
					Spinal cord in	Spina bifid
59	M	150	45	20	0	0
58	M	172	56	18.9	1	0
15	M	140	46	23.46	0	1
42	M	168	60	21.25	1	0
71	M	169	70	24.5	1	0
46	M	169	60	21	1	0
60	M	171	66	22.57	1	0
84	M	165	60	22.03	1	0
63	M	170	75	25.95	1	0
59	M	162	63	24	1	0
48	M	174	82	27.08	1	0
35	F	164	—	—	1	0
33	M	157	53	21.5	0	1
17	M	160	50	19.53	0	1
43	M	180	70	21.6	1	0
21	M	150	62	27.55	0	1
61	M	180	56	17.28	1	0
29	M	168	57	20.19	1	0
54	M	173	60	20.04	1	0
63	M	176	68	21.95	1	0
51	M	170	59.7	20.65	1	0
54	M	173	—	—	1	0
68	M	170	65	22.49	0	0
61	M	174	70.1	23.15	1	0
35	M	143	60	29.34	0	1
47	M	183	90	26.87	1	0
69	M	162	65	24.76	1	0
	M：N=26				N=20	N=5
	F：N= 1					

55	M	—	—	—	1	0
37	M	170	64	22.14	1	0
56	M	168.5	60	21.13	1	0
57	M	165	65	23.87	1	0
42	M	170	71	24.56	1	0
63	M	174	68	22.46	1	0
59	M	161	53	20.44	0	0

57	M	170	65	22.49	1	0
68	M	168	47	16.65	0	0
39	M	166	78	28.3	1	0
51	M	170	60	20.76	1	0
	M:N=1 1				N=9	N=0
	F:N=0					

ase	Duration of wheelchair mar	of primary diseas		dominant hand
polio	others			
0	1	25	59	R
0	0	32	36	R
0	0	7	15	R
0	0	4	10	R
0	0	25	41	R
0	0	8	12	R
0	0	10	13	R
0	0	31	38	R
0	0	18	29	L
0	0	7	8	R
0	0	5	20	R
0	0	7	20	R
0	0	18	33	R
0	0	5	17	R
0	0	18	18	R
0	0	3	21	R
0	0	23	48	L
0	0	3	4	L
0	0	29	35	R
0	0	2	28	R
0	0	18	20	R
0	0	4	30	R
0	on of both lo	24	48	R
0	0	38	40	L
0	0	20	35	R
0	0	28	29	R
0	0	34	38	R
N=	N=2			Right ：N=23
				Left ：N=4

0	0	20.5	36	R
0	0	12	12	R
0	0	24	29	L
0	0	29	34	R
0	0	15	19	R
0	0	8	18	R
1	0	20	58	R

0	0	16	28	R
1	0	38	68	R
0	0	4	8	R
0	0	30	44	R
N=2	N=0			Right ：N=10
				Left ：N=1

ining time(hours/sessi	ing frequency(times/w	training frequency(ti	y of other wheelchair s
			+
2	2	0	0
3	0.5	0	1
2	2	0	0
5	2	3≧	0
1	1	0	1
2	1.5	0	0
4	1	2	0
4	1.5	2	1
4	2	1	0
7	1	3≧	1
3	1.5	0	0
1	0.5	3≧	1
6	1.5	0	0
3	2	0	0
2	0.5	0	1
5	3	0	1
1	1.5	0	0
6	2	2	0
2	2	0	1
2	1	0	1
1.5	1.5	0	0
5	1.5	0	1
3	1.5	0	0
1	1	3≧	1
2	1.5	0	0
2.5	1	0	0
5	1	0	1
		1：N＝1	N=12
		2：N＝3	
		3≧：N＝4	
2	2	0	0
2	2.5	1	0
7	1	3≧	0
0.75	―	0	0
2	2	0	0
2	1.5	0	0
3	1.5	0	0

2	1.5	2	0
2	1	0	1
3	1.5	0	0
—	—	—	0
		1:N=1<br>2:N=1<br>3≧：N＝1	N=1

y of wheelchair sports	US: dislocation	
+	Rt	Lt
0	0	0
0	0	0
0	0	0
0	0	0
0	0	0
0	0	0
0	0	0
1	0	0
0	0	0
0	0	0
1	0	0
1	0	0
0	0	0
1	1	0
0	0	0.5
0	0	0
0	0	0
0	0	0
1	0	0
0	0	0.5
1	0	0.5
0	0	0
0	0.5	0
1	0.5	0
0	0	1
1	0	0.5
1	0	0
N= 9	1:complete N=1	N=1
	0.5:partial N=2	N=4

1	0	0
0	1	0
0	0	0
0	1	0.5
0	0	0
0	0.5	0.5
0	1	0.5

1	0	0
1	1	1
0	1	1
0	0	1
N=3	N=5	N=3
	N=1	N=3
