# Supplementary material for: The prevalence of ulnar neuropathy at the elbow and ulnar nerve dislocation in recreational wheelchair marathon athletes
Source: PLoS One. 2020 Dec 14;15(12):e0243324. doi: 10.1371/journal.pone.0243324 (PMC7735619; doi:10.1371/journal.pone.0243324)
Supplement: S2 Table — (PDF) [file pone.0243324.s004.pdf]

Numness	Ring split sign	Tinel sign	Froment sign	paralysis /	paralysis /	dislocatior
RT	Rt	Rt		Rt	Rt	Rt
0	0	0	0	0	0	0
1	0	0	0	0	0	1
0	0	0	0	0	0	0
0	0	0	0	0	0	1
0	—	1	0	0	0	0
0	—	1	0	0	0	0.5
0	1	1	0	0	0	1
0	0	0	0	0	0	0
0	1	1	0	0	0	1
0	0	1	0	0	0	1
1	0	1	0	0	0	0

Numness Lt	Ring split Lt	Tinel sign Lt	Froment s	paralysis Lt	paralysis Lt	dislocatior Lt
1	0	1	0	0	0	0
0	0	0	0	0	0	0
0	1	1	0	0	0	0
0	0	1	0	0	0	0.5
0	—	0	0	0	0	0
0	—	0	0	0	0	0.5
0	0	0	0	0	0	0.5
1	0	1	0	0	0	0
0	0	1	0	0	0	1
0	0	1	0	0	0	1
1	0	1	0	0	0	1

dominant hand
R
R
L
R
R
R
R
R
R
R
R
